# COMMUNITY INTEGRATION AND ITS PREDICTORS IN PEOPLE WITH STROKE: A MULTICENTER LONGITUDINAL STUDY

**DOI:** 10.2340/jrm.v56.21372

**Published:** 2024-04-25

**Authors:** Jiang-Li ZHAO, Lian-Dong MA, Xiang XIAO, Li-Jun LIN, Hao XIE, Shamay S. M. NG, Pei-Ming CHEN

**Affiliations:** 1 Department of Rehabilitation Medicine, The First Affiliated Hospital, Sun Yat-sen University, Guangzhou, China; 2 Department of Acupuncture and Rehabilitation Medicine, The Second Traditional Chinese Medicine Hospital, Guangzhou, China; 3 Department of Rehabilitation Medicine, The Shenzhen Luohu Hospital Group Luohu People’s Hospital, The Third Affiliated Hospital of Shenzhen University, Shenzhen, China; 4 Department of Rehabilitation Sciences, The Hong Kong Polytechnic University, Hong Kong SAR, China

**Keywords:** stroke, rehabilitation, community integration, community integration questionnaire, balance function, daily living ability

## Abstract

**Objective:**

To investigate the community integration of patients following stroke and determine the predictors of their level of community integration at 1-year follow-up.

**Design:**

A multicenter, longitudinal, and observational study.

**Subjects:**

Sixty-five inpatients (41 men) with a mean age of 56.9 (standard deviation = 17.0) years, who had their first stroke at least 1 month prior to this study were recruited from 4 rehabilitation inpatient wards in China.

**Methods:**

In the initial assessment, the participants were evaluated using the Community Integration Questionnaire, the Fugl-Meyer Assessment, the Berg Balance Scale, the Modified Barthel Index, the Mini Mental State Examination, and the Modified Ashworth Scale. In the follow-up assessments, which were conducted via telephone no less than 1 year after discharge, the participants were evaluated using the Community Integration Questionnaire and also assessed for other disease-related conditions.

**Results:**

The participants’ scores on the Community Integration Questionnaire in the follow-up assessment were significantly greater than those at the initial assessment (*p* < 0.05). In addition, the participants’ Community Integration Questionnaire scores in the follow-up assessment were significantly correlated with their ages, numbers of years of education, and Modified Barthel Index, Berg Balance Scale, Mini Mental State Examination scores in the initial assessment (*p* < 0.05), and marginally significantly correlated with their scores on Fugl-Meyer Assessment in the initial assessment (*p* = 0.058). The participants’ ages, numbers of years of education, and Modified Barthel Index, Berg Balance Scale, Mini Mental State Examination, Fugl-Meyer Assessment of the lower extremity, and Fugl-Meyer Assessment scores in the initial assessment were predictive of their Community Integration Questionnaire scores at follow-up, with coefficients of determination ranging from 0.254 to 0.056 (*p* < 0.05).

**Conclusions:**

The level of community integration of the participants was generally low, but it was greater at 1-year follow-up than it was initially. Balance function and daily living ability may be key predictors of community integration of patients following stroke.

LAY ABSTRACT

Many stroke survivors experience persistent motor impairment, which directly impacts their ability to perform daily activities and makes it difficult for them to integrate into communities. In addition, stroke rehabilitation programmes may be only partially effective and are often ineffective. This study investigated the community integration of patients following stroke and explored its predictors to provide a basis for the development of stroke rehabilitation programmes that can improve the quality of life of patients and enable them to achieve community integration. The results showed that balance function, age, numbers of years of education, cognitive function, limb motor function, and ability to perform activities of daily living at the initial assessment were predictive of the level of community integration at 1-year follow-up. Balance function and ability to perform activities of daily living appear to be key predictors of community integration in people with stroke. Therefore, focusing stroke rehabilitation on improving balance and the ability to perform activities of daily living may increase the community integration of patients recovering from a stroke.

Stroke is a significant contributor to mortality and disability worldwide (1). In addition, as the world’s population continues to grow and age, the number of stroke survivors is increasing (2). Following a stroke, approximately 33% of patients experience persistent motor impairment (3), and between 15% and 30% of patients are permanently disabled (4). Motor impairment following a stroke has a direct and adverse impact on patients’ abilities to perform daily living activities, negatively affects their quality of life, and places a heavy economic burden on their family and on society (5).

The ultimate goal of rehabilitation after traumatic brain injury is to enable patients to achieve community integration (CI) (6). Similarly, patients with stroke experience motor and cognitive impairment, which can have a substantial impact on their level of CI in daily life. Willer et al. (7) defined CI as integration into family-like communities and social networks and involvement in productive activities, such as work, school, or volunteering.

The Community Integration Questionnaire (CIQ) is used to evaluate the effectiveness of rehabilitation programmes for patients with traumatic brain injury (7). It is composed of three subscales: a Home Integration (HI) subscale, a Social Integration (SI) subscale, and a Productive Activity (PA) subscale. The CIQ has been proven to be reliable and valid for assessing various patient populations (8), and a Chinese version of the CIQ has been developed and validated for use in Chinese patients (9).

Many patients with stroke experience moderate to severe disability (10, 11), which can impede their ability to resume daily activities and return to their previous lifestyle, thereby hindering their ability to socially integrate into their communities (12, 13). Therefore, increasing the CI of patients with stroke is the ultimate objective of stroke rehabilitation, which means that identifying a sensitive indicator of CI is crucial for optimizing stroke rehabilitation. To the best of our knowledge, no study has been performed in China to investigate the level of CI in patients with stroke. Furthermore, low levels of social participation and poor quality of life remain significant problems for people with stroke worldwide (8).

This study used the CIQ to assess the CI of Chinese patients with stroke, evaluating their cognitive impairments, motor impairments, balance impairments, and self-care ability in daily living (ADL). The objective of this study was to identify the significant predictors of CIQ scores and investigate the strength of these predictors in people with stroke. The results of this study can serve as a basis for the development of stroke rehabilitation programmeffs that can improve the quality of life of patients with stroke, optimize the allocation of medical resources, and reduce economic burdens on families and societies.

## MATERIALS AND METHODS

### Study design

This was a multicentre, longitudinal, observational study that was conducted in 3 hospitals across 4 centres in China (Table I). The participants’ demographic information and major comorbidity data were obtained from their medical records. The study was approved by the Human Subjects Ethics Subcommittee of the First Affiliated Hospital of Sun Yat-sen University in China. Written informed consent was obtained from all participants prior to their assessment.

**Table I:** Collection status of effective scales in each centre

Centre	Valid questionnaires *n (%)*
Department of Rehabilitation Medicine, The First Affiliated Hospital of Sun Yat-sen University	16 (24.6)
Department of Acupuncture and Rehabilitation, Guangdong Second Traditional Chinese Medicine Hospital Branch	29 (44.6)
Department of Acupuncture and Rehabilitation, Guangdong Second Traditional Chinese Medicine Hospital	7 (10.8)
Department of Rehabilitation Medicine, Shenzhen Luohu Hospital Group Luohu People’s Hospital, The Third Affiliated Hospital of Shenzhen University	13 (20)
Total	65 (100)

### Sample size calculation

The sample size was determined based on a previous study that investigated the factors contributing to CI of patients with aphasia after stroke (8). In that study, we found that a minimal sample size of 30 was sufficient to detect a significant correlation between Stroke and Aphasia Quality of Life Scale-39 scores and CIQ scores in patients with stroke. In contrast, in this study, we increased the sample size to 65 to enable more robust conclusion to be drawn.

### Participants

Potential participants were recruited from July to December 2020 and were included in this study if (*i*) they had experienced a first stroke with unilateral hemiparetic lesions confirmed by magnetic resonance imaging or computed tomography; (*ii*) their stroke had occurred at least 1 month prior; (*iii*) they had no severe deficits in communication; and (*iv*) they were able to give informed consent. Potential participants were excluded from the study if they (*i*) were unable to complete the assessments due to medical instability; (*ii*) has been diagnosed with other neurological diseases that may affect cognitive function; (*iii*) had psychological diseases not caused by stroke; (*iv*) had aphasia or were unable to communicate; (*v*) had a limb fracture; or (*vi*) they or their families did not agree to their participation in the study.

### Study protocol

Prior to the collection of baseline data, each centre was staffed with a therapist with over 10 years of clinical experience, who oversaw the screening, questionnaires-based assessment, and functional assessment of patients. All raters underwent training on the proper administration of outcome measures based on recent guidelines. Demographic data (e.g. gender, age, education, mobility, and stroke type) and the stroke-specific outcome measures (i.e. the FMA, BBS, MBI, MMSE, and MAS) were collected, as these are factors that may affect the level of CI of people with stroke. Written or oral informed consent was obtained from the participants before the initial assessment, which took approximately 1 hour, with sufficient rest periods provided to prevent fatigue. Follow-up assessments were conducted 1 year later via telephone to determine the participants’ then-current CIQ score, disease recurrences, and numbers of falls in the past year.

### Outcome measures

*Community Integration Questionnaire (CIQ).* The CIQ was originally designed for assessing people with a traumatic brain injury (14). It is a brief, reliable (intraclass correlation coefficients [ICC] = 0.69] measure of a person’s level of CI (7). It comprises 15 questions that assess a person’s ability to perform various activities. The responses for questions 1–10 and 12 are rated on a 3-point scale (0, 1, or 2), whereas the response for question 11 is rated on a 2-point scale (0 or 2). The responses to questions 13–15 are combined to create a single variable that is scored from 0 to 5 points. As mentioned, the CIQ comprises 3 subscales, namely an HI subscale (comprising questions 1–5 and with a total possible score ranging from 0 to 10 points), an SI subscale (comprising questions 6–11 and with a total possible score ranging from 0 to 12 points), and a PA subscale (comprising questions 12–15 and with a total possible score ranging from 0 to 7 points). The total possible score on the CIQ ranges from 0 to 29 points, with higher scores indicating better CI. The CIQ is typically self-administered, but if the person being assessed is unable to do this, someone familiar with the person may complete the CIQ on the person’s behalf while the person is present.

*Fugl-Meyer Motor Assessment (FMA).* The FMA, devised by Twitchell and Brunnstrom, was the first quantitative evaluative instrument developed to measure sensorimotor stroke recovery, and is based on the concept of sequential stages of motor return in hemiplegic patients with stroke (15). The motor domain of the FMA is divided into two sessions: an upper-extremity (UE) section (FMA-UE) and a lower-extremity (LE) session (FMA-LE), which consist of 33 and 17 items, respectively. Each item is scored on a 3-point ordinal scale, where 0 = could not perform, 1 = partially performed, and 2 = fully performed. The total possible score on the FMA-UE ranges from 0 to 66 points, the total possible score on the FMA-LE ranges from 0 to 34 points, and the total possible score on the FMA ranges from 0 to 100 points, with higher scores indicating less motor impairment. The FMA has been widely used and has good-to-excellent inter-rater reliability (overall score, ICC = 0.98; FMA-UE score, ICC = 0.99; FMA-LE score, ICC = 0.91) and intra-rater reliability (overall score, ICC = 0.99; FMA-UE score, ICC = 0.95; FMA-LE score, ICC = 0.99) (16).

*Berg Balance Scale (BBS).* The BBS is an assessment tool that is widely used in stroke rehabilitation and comprises 14 items, each of which is scored on a scale from 0 to 4 points (17). The total possible score on the BBS ranges from 0 to 56 points, with higher scores indicating better balance function, and scores of less than 40 points indicating a risk of falling. The BBS has demonstrated reliability and consistency, with high internal consistency (Cronbach’s alpha = 0.92–0.98), inter-rater reliability (ICC = 0.95–0.98), intra-rater reliability (ICC = 0.97), and test–retest reliability (ICC = 0.98) in assessing balance in patients with stroke (17, 18).

*Modified Barthel Index (MBI).* The MBI is a commonly used tool for assessing patients’ self-care ADL that reflects functional capacity, particularly in patients with stroke (19). It consists of 10 items, with the total possible score ranging from 0 to 100 points, and a higher score indicates a better ADL. The MBI has demonstrated excellent reliability in assessing ADL in patients with stroke (ICC = 0.88) (20).

*Mini Mental State Examination (MMSE).* The MMSE is a commonly used tool for assessing cognitive function in patients with stroke. It comprises 30 items that assess orientation, memory, calculation, recall, and language (21). The total possible score of the MMSE ranges from 0 to 30 points. The MMSE has demonstrated excellent reliability (ICC = 0.94) in assessing cognitive function (22).

*Modified Ashworth Scale (MAS).* The MAS is a commonly used tool for assessing spasticity in patients with stroke and quantifies the severity of spasticity as grade 0, 1, 1+, 2, 3, or 4. These grades were allocated 0, 1, 1.5, 2, 3, and 4 points respectively, in the current study, which assessed the spasticity of the flexors of the elbow, wrist, and finger, and the extensors of the knee and ankle. The MAS has demonstrated excellent inter-rater reliability (ICC = 0.686–0.781) and intra-rater reliability (ICC = 0.644–0.748) in assessing spasticity in patients (23).

### Statistical analysis

Descriptive statistics are used to present the demographic and clinical characteristics of the participants. A one-sample Kolmogorov–Smirnov test was used to test whether the data were normally distributed. The FMA-LE scores of all the included participants; the age, and the initial FMA-LE and BBS scores of those who completed the follow-up assessment; and the age, numbers of years of education, and initial FMA-UE, FMA-LE, FMA, and CIQ-HI scores of those who withdrew from the study were normally distributed, whereas all other data were non-normally distributed. Independent sample *t*-tests, Mann–Whitney *U* tests, and χ^2^ tests were employed to identify any significant differences between participants who completed the follow-up assessment and those who withdrew from the study. Wilcoxon rank-sum tests were employed to identify significant differences in the overall mean scores at the 2 assessment time points (baseline and follow-up) for the participants who completed the follow-up assessment. At baseline, Kruskal–Wallis tests and Mann–Whitney *U* tests were employed to identify significant differences in the overall medians of 2 or more independent samples grouped by various factors, such as gender, stroke type, hemiplegic side, move mode, and occupation. Spearman correlation coefficients (ρ) were calculated to assess correlations between non-parametric data. A univariate linear regression model was used in the “enter” method to analyse the linear quantitative relationships between related factors and CIQ scores.

All statistical analyses were conducted using SPSS version 20.0 (IBM Corp, Armonk, NY, USA), and all tests were two-tailed with a *p*-value of less than 0.05 regarded as indicating a significant difference.

## RESULTS

### Demographics

Sixty-five people (41 men and 24 women) who had experienced a first stroke were enrolled from 4 departments of rehabilitation medicine in 3 hospitals from July to December 2020. Table I presents the details of the participants from each of the four centres.

The participants had a mean age of 56.9 years (standard deviation (SD) = 17.0 years; range = 14–90 years) and had experienced their first stroke a mean of 9.6 months (SD = 12.6 months; range = 1–60 months) ago. One year after the initial assessment, 57 participants completed a follow-up assessment via telephone. Among these participants, 5 had recurrent illness, 14 had a history of falls, 8 had returned to work or study, and 16 had not reached retirement age but had not yet returned to work. The reasons for withdrawal were death (2 participants), refusal to participate in the telephone follow-up (1 participant), and lost to follow-up (5 participants).

There were no statistically significant differences between the follow-up group and the withdrawal group in terms of age, post-stroke duration, numbers of years of education, gender, marital status, stroke type, affected side, move mode, and occupation. Table II provides details on all of the participants (*n* = 65), those who withdrew (*n* = 8), and those who completed the follow-up assessment (*n* = 57).

**Table II:** Characteristics of study participants

Factor	Total (*n* = 65)	Complete (*n* = 57)	Withdrew (*n* = 8)
Age (years)^a^	56.9 (17.0)	57.0 (16.2)	56.3 (23.6)
Onset (months)^b^	5.0 (3.0, 8.0)	5.0 (3.0, 8.5)	3.5 (3.0, 6.8)
Education (years)^b^	11.0 (8.0, 14.0)	11.0 (8.0, 14.0)	11.0 (8.8, 14.8)
Sex			
Male	41 (63.1)	36 (63.2)	5 (62.5)
Female	24 (36.9)	21 (36.8)	3 (37.5)
Marital status			
Married	61 (93.8)	54 (94.7)	7 (87.5)
Unmarried	3 (4.6)	2 (3.5)	1 (12.5)
Divorce	1 (1.5)	1 (1.8)	0 (0)
Stroke type			
Haemorrhage	24 (36.9)	20 (35.1)	4 (50)
Infarction	41 (63.1)	37 (64.9)	4 (50)
Affected side			
Right	27 (41.5)	24 (42.1)	3 (37.5)
Left	35 (53.8)	31 (54.4)	4 (50)
Bilateral	3 (4.6)	2 (3.5)	1 (12.5)
Move mode			
Walk	33 (50.8)	27 (47.4)	6 (75)
combined	4 (43.1)	4 (7.0)	0 (0)
Wheelchair	28 (6.2)	26 (45.6)	2 (25)
Occupation			
Employed	31 (47.7)	28 (49.1)	3 (37.5)
Retired	31 (47.7)	26 (45.6)	5 (62.5)
Unemployed	3 (4.6)	3 (5.3)	0 (0)

Values are mean (SD), median (25^th^ percentile, 75^th^ percentile) or *n* (%).

a Independent sample *t*-test.

b Mann–Whitney *U* test.

*p < 0.05 indicates significant correlations between withdrew and complete group.

### Community Integration Questionnaire scores and other outcome measures data

Table III presents the CIQ scores and other outcome measures data of all of the included participants, the completion group (which comprised the participants who completed both the assessments, i.e. the initial and follow-up assessment), and the withdrawal group. The results indicated that there were no statistically significant between-group differences in CIQ, MAS, MMSE, FMA-UE, FMA-LE, FMA, BBS, or MBI scores (*p* > 0.05). Within the completion group, the scores differed significantly between the initial and follow-up assessments for the CIQ, CIQ-HI, and CIQ-PA (*p* < 0.05) but not for the CIQ-SI (*p* > 0.05). These differences suggest that the complete group had become integrated into their families and increased their productivity during the 1-year period that ended in follow-up.

**Table III:** Scores of outcome measures in study participants

Variable	Total (*n* = 65)	Withdrew (*n* = 8)	Complete (initial) (*n* = 57)	Complete (follow-up) (*n* = 57)	*p*_1_	*p*_2_
CIQ	5.00 (3.0, 11.8)	6.75 (4.0, 16.8)	4.25 (3.0, 11.3)	6.00 (3.0, 19.4)	0.261^a^	0.005**^c^
HI	0.00 (0.0, 4.0)	1.75 (0.0, 8.3)	0.00 (0.0, 3.5)	1.00 (0.0, 7.8)	0.263^a^	0.004**^c^
SI	4.00 (2.5,8.0)	5.00 (4.0, 8.0)	4.00 (2.0, 7.5)	4.00 (2.0, 10.0)	0.255^a^	0.206^c^
PA	0.00 (0.0, 0.0)	0.00 (0.0, 0.0)	0.00 (0.0, 0.0)	0.00 (0.0, 2.0)	0.340^a^	0.000**^c^
MMSE	27.00 (22.0, 30.0)	29.50 (26.3, 30.0)	27.00 (19.5, 30.0)		0.162^a^	
MAS	1.00 (0.0, 3.0)	0.75 (0.0, 1.9)	1.00 (0.0, 3.0)		0.377^a^	
FMA-UE	31.00 (11.5, 60.0)	34.50 (19.0, 62.5)	30.00 (10.5, 59.0)		0.484^a^	
FMA-LE	21.46 (8.81)	23.63 (8.94)	21.16 (8.83)		0.463^b^	
FMA	51.00 (24.0, 89.0)	54.50 (37.8, 94.3)	51.00 (23.5, 88.5)		0.430^a^	
BBS	33.00 (22.5, 45.5)	38.00 (12.5, 48.0)	32.00 (22.5,45.0)		0.442^a^	
MBI	75.00 (50.0, 92.5)	85.00 (57.8, 97.5)	70.00 (50.0, 92.5)		0.279^a^	

Values are mean (SD) or median (25^th^ percentile, 75^th^ percentile).

a Mann–Whitney U test.

b Independent sample *t*-test.

c Wilcoxon Rank-sum test.

*p* < 0.05 indicates significant difference. *p < 0.05.

** *p* < 0.01. *p*_1_ was the *p*-value of comparison between withdrew (*n* = 8) and complete (*n* = 57) group. *p*_2_ was the *p*-value of comparison between initial and follow-up assessment of CIQ score in the complete group (*n* = 57).

CIQ: Community Integration Questionnaire; HI: Home Integration of Community Integration Questionnaire; SI: Social Integration of Community Integration Questionnaire; PA: Productivity Activity of Community Integration Questionnaire; MMSE: Mini Mental State Examination; MAS: Modify Ashworth Scale; FMA-UE: Upper Extremity section of the Fugl–Meyer Assessment; FMA-LE: Lower Extremity section of the Fugl–Meyer Assessment; BBS: Berg Balance Scale; MBI: Modified Barthel Index.

### Analysis of Community Integration Questionnaire scores grouped by classification index

Table IV shows the initial CIQ scores of all of the total participants grouped by and compared using various factors. The results indicate that compared with the walk group and the walk + wheelchair group, the wheelchair group obtained lower scores on the CIQ, CIQ-HI, and CIQ-SI (*p* < 0.05). Moreover, the retired group had a lower CIQ-PA score than the employed and unemployed groups (*p* < 0.05).

**Table IV:** Scores of Community Integration Questionnaire (CIQ) in initial assessment grouped and compared by different factors in the stroke participants (*n* = 65)

Factor	Sex^a^		Stroke type^a^		Move mode^b^			Occupation^b^		
	Male	Female	Haemorrhage	Infarction	Walk	Combined	Wheelchair	Employed	Retired	Unemployed
Number	41	24	24	41	33	4	28	31	31	3
CIQ, median (IQR)	4.25 (3.0, 10.0)	5.00 (4.0, 11.9)	4.13 (3.0, 7.4)	5.00 (3.0, 14.5)	8.00 (4.0, 16.6)	5.63 (3.1, 12.5)	3.00 (2.0, 5.0)**	4.00 (3.0, 7.0)	6.00 (4.0, 14.0)	6.00 (2.0, –)
HI, median (IQR)	0.00 (0.0, 3.5)	0.00 (0.0, 4.8)	0.00 (0.0, 2.4)	0.00 (0.0, 5.5)	2.50 (0.0, 6.6)	2.13 (0.3, 3.0)	0.00 (0.0, 0.0)**	0.00 (0.0, 1.3)	1.25 (0.0, 5.0)	0.00 (0.0, –)
SI, median (IQR)	4.00 (2.0, 7.5)	4.50 (3.3, 8.8)	4.00 (2.0, 5.0)	4.00 (3.0, 8.5)	5.00 (4.0, 9.0)	4.00 (2.3, 7.3)	3.00 (2.0, 5.0)**	4.00 (2.0, 6.0)	4.00 (3.0, 9.0)	6.00 (2.0, –)
PA, median (IQR)	0.00 (0.0, 0.0)	0.00 (0.0, 0.0)	0.00 (0.0, 0.0)	0.00 (0.0, 0.0)	0.00 (0.0, 0.0)	0.00 (0.0, 2.3)	0.00 (0.0, 0.0)	0.00 (0.0, 0.0)	0.00 (0.0, 0.0)**	0.00 (0.0, 0.0)

a Mann–Whitney *U* test.

b Kruskal–Wallis test.

*p* < 0.05 indicates significant correlations. *p < 0.05.

** *p* < 0.01.

IQR: interquartile range; HI: Home Integration of Community Integration Questionnaire; SI: Social Integration of Community Integration Questionnaire; PA: Productivity Activity of Community Integration Questionnaire.

### Correlations between Community Integration Questionnaire scores and initial outcome measures

Table V presents the details of correlations between the participants’ CIQ scores and other outcome measures in the initial assessment. The results indicate that there were statistically significant correlations between the participants’ CIQ scores and their ages, MMSE scores, FMA-UE scores, FMA-LE scores, FMA scores, BBS scores, and MBI scores (*p* < 0.05). This suggests that their CIQ scores were influenced by their ages, cognitive function, motor function, balance function and ADL, with the latter two factors being particularly influential.

**Table V:** Correlations between Community Integration Questionnaire (CIQ) and other outcome measures in initial evaluation of all stroke participants (*n* = 65)

Factor	CIQ		HI		SI		PA	
	ρ	*p*	ρ	*p*	ρ	*p*	ρ	*p*
Age	–0.348**	0.004	–0.377**	0.002	–0.243	0.051	–0.264*	0.033
Education	0.223	0.074	0.406**	0.001	0.171	0.174	0.306*	0.013
Onset	0.100	0.428	0.145	0.249	0.067	0.597	–0.015	0.906
MAS	–0.151	0.231	–0.020	0.874	–0.179	0.153	–0.228	0.067
MMSE	0.500**	0.000	0.472**	0.000	0.497**	0.000	0.203	0.105
FMA-UE	0.308*	0.012	0.247*	0.048	0.227	0.069	0.317**	0.010
FMA-LE	0.467**	0.000	0.461**	0.000	0.358**	0.003	0.360**	0.003
FMA	0.388**	0.001	0.338**	0.006	0.300*	0.015	0.353**	0.004
BBS	0.611**	0.000	0.590**	0.000	0.487**	0.000	0.364**	0.003
MBI	0.652**	0.000	0.672**	0.000	0.486**	0.000	0.378**	0.002

*p* < 0.05 indicates significant correlations.

* *p* < 0.05.

** *p* < 0.01.

HI: Home Integration of Community Integration Questionnaire; SI: Social Integration of Community Integration Questionnaire; PA: Productivity Activity of Community Integration Questionnaire; MMSE: Mini Mental State Examination; MAS: Modified Ashworth Scale; FMA-UE: Upper Extremity section of the Fugl–Meyer Assessment; FMA-LE: Lower Extremity section of the Fugl–Meyer Assessment; BBS: Berg Balance Scale; MBI: Modified Barthel Index.

Table VI displays the details of the correlations between the participants’ CIQ in the follow-up assessment and their CIQ scores and other outcome measures in the initial assessment. The results indicate that their CIQ scores in the follow-up assessment were significantly correlated with their CIQ scores, ages, numbers of years of education, MMSE scores, BBS scores, and MBI scores in the initial assessment (*p* < 0.05), suggesting that these factors continued to affect their level of CI 1 year after the initial assessment.

**Table VI:** Correlations between follow-up Community Integration Questionnaire (CIQ) with initial outcome measures in follow-up participants (*n* = 57)

Factor	CIQ (follow-up)		HI (follow-up)		SI (follow-up)		PA (follow-up)	
	ρ	*p*	ρ	*p*	ρ	*p*	ρ	*p*
Age	–0.497**	0.000	–0.493**	0.000	–0.430**	0.001	–0.433**	0.001
Education	0.309*	0.019	0.373**	0.004	0.247	0.064	0.347**	0.008
Onset	–0.057	0.674	0.098	0.470	–0.147	0.257	0.040	0.767
MAS	0.026	0.848	0.034	0.801	–0.011	0.935	0.091	0.502
MMSE	0.383**	0.003	0.371**	0.004	0.366**	0.005	0.304*	0.021
FMA-UE	0.246	0.065	0.301*	0.023	0.198	0.140	0.190	0.156
FMA-LE	0.257	0.054	0.300*	0.023	0.196	0.144	0.166	0.218
FMA	0.252	0.058	0.301*	0.023	0.213	0.112	0.188	0.161
BBS	0.516**	0.000	0.502**	0.000	0.435**	0.001	0.324*	0.014
MBI	0.564**	0.000	0.544**	0.000	0.491**	0.000	0.409**	0.002
CIQ (initial)	0.562**	0.000	0.523**	0.000	0.489**	0.000	0.473**	0.000
HI (initial)	0.557**	0.000	0.560**	0.000	0.500**	0.000	0.444**	0.001
SI (initial)	0.437**	0.001	0.391**	0.003	0.396**	0.002	0.375**	0.004
PA (initial)	0.504**	0.000	0.515**	0.000	0.459**	0.000	0.430**	0.000

*p* <0.05 indicates significant correlations.

* *p* < 0.05.

** *p* < 0.01.

HI: Home Integration of Community Integration Questionnaire; SI: Social Integration of Community Integration Questionnaire; PA: Productivity Activity of Community Integration Questionnaire; MMSE: Mini Mental State Examination; MAS: Modified Ashworth Scale; FMA-UE: Upper Extremity section of the Fugl–Meyer Assessment; FMA-LE: Lower Extremity section of the Fugl–Meyer Assessment; BBS: Berg Balance Scale; MBI: Modified Barthel Index.

### Factors that predicted follow-up Community Integration Questionnaire scores in the linear regression model

The results showed that the MBI scores, BBS scores, age, numbers of years of education, MMSE scores, FMA-LE scores, and FMA scores in the initial assessment were good predictors of the follow-up CIQ scores, as the corresponding adjusted R^2^ values ranged from 0.254 to 0.056 (*p* < 0.05). In addition, the initial FMA-UE score was a fair predictor of the follow-up CIQ-HI score (adjusted *R*^2^ = 0.070, *p* < 0.05), and the initial CIQ-HI score was the best predictor of the follow-up CIQ, CIQ-HI, CIQ-SI, and CIQ-PA scores. After the initial CIQ scores, BBS and MBI scores were the best predictors of the follow-up CIQ, CIQ-HI, and CIQ-SI scores. However, numbers of years of education was the best predictor of the follow-up CIQ-PA score. More information on the linear regression results can be found in Table VII.

**Table VII:** Univariate linear regression of follow-up Community Integration Questionnaire (CIQ) and other initial outcome measures in follow-up participants (*n* = 57)

Factor	CIQ (follow-up)			HI (follow-up)			SI (follow-up)			PA (follow-up)		
	Β	β	Adjusted R^2^	Β	β	Adjusted R^2^	Β	β	Adjusted R^2^	Β	β	Adjusted R^2^
Age	–0.252	–0.462**	0.199	–0.102	–0.409**	0.152	–0.108	–457**	0.194	–0.042	-0.399**	0.144
Education	0.763	0.352**	0.108	0.332	0.336*	0.097	0.260	0.275*	0.059	0.171	0.412**	0.155
MMSE	0.369	0.326*	0.090	0.153	0.297*	0.072	0.163	0.329*	0.092	0.053	0.245	0.043
FMA-UE	0.094	0.249	0.045	0.051	0.294*	0.070	0.030	0.182	0.016	0.013	0.186	0.017
FMA-LE	0.284	0.284*	0.064	0.147	0.324*	0.088	0.095	0.219	0.031	0.041	0.214	0.028
FMA	0.077	0.269*	0.056	0.041	0.315*	0.083	0.025	0.200	0.023	0.011	0.202	0.023
BBS	0.264	0.505**	0.242	0.118	0.495**	0.231	0.109	0.480**	0.216	0.037	0.368**	0.119
MBI	0.178	0.517**	0.254	0.080	0.507**	0.243	0.072	0.482**	0.219	0.026	0.396**	0.142
CIQ (initial)	0.860	0.618**	0.371	0.374	0.589**	0.335	0.337	0.556**	0.296	0.149	0.561**	0.302
HI (initial)	1.667	0.582**	0.327	0.758	0.580**	0.325	0.647	0.518**	0.255	0.263	0.479**	0.216
SI (initial)	1.343	0.489**	0.225	0.556	0.443**	0.182	0.548	0.457**	0.195	0.239	0.455**	0.193
PA (initial)	4.873	0.554**	0.294	2.093	0.552**	0.259	1.748	0.456**	0.193	1.033	0.613**	0.365

*p* < 0.05 indicates significant correlations.

* *p* < 0.05.

** *p* < 0.01.

B: Unstandardized beta; β: Standardized beta; HI: Home Integration of Community Integration Questionnaire; SI: Social Integration of Community Integration Questionnaire; PA: Productivity Activity of Community Integration Questionnaire; MMSE: Mini Mental State Examination; MAS: Modified Ashworth Scale; FMA-UE: Upper Extremity section of the Fugl–Meyer Assessment; FMA-LE: Lower Extremity section of the Fugl–Meyer Assessment; BBS: Berg Balance Scale; MBI: Modified Barthel Index.

## DISCUSSION

This study was the first to examine the relationship between CI and other stroke-specific outcomes in patients with stroke in China. Our study demonstrated that age, numbers of years of education, cognitive function, upper- and lower- limb motor function, body balance function, and self-care ADL were factors that affected the CI levels of the participants following stroke. Moreover, the CI of the participants was greater at 1-year follow-up than at the initial assessment, and self-care ADL, balance function, age, numbers of years of education, cognitive function, and limb motor function in the initial assessment were good predictors of the CI level in the 1-year follow-up assessment. Furthermore, the influence of motor function on CI decreased over time.

### Level of community integration

The mean CIQ score was 7.55 (SD = 6.32), which is comparable to the mean CIQ scores of patients in South Korea with aphasia following stroke, as reported by Lee et al. (8) (8.5 [SD = 5.3]). This indicates that the participants in the current study had low levels of CI. Similarly, the participants had low CIQ-HI, CIQ-SI, and CIQ-PA scores.

However, unlike the participants in the current study, Lee et al. (8) reported that patients with aphasia following stroke did not significantly reduce the amount of time they spent doing indoor activities, such as cooking and housework. This may be due to the fact that Lee et al. included only patients who were able to independently perform daily activities and move around, whereas only 50% (33/65) of the participants in the current study could walk, and most did not have self-care ADL. Moreover, due to the differences between the food cultures of China and South Korea, cooking maybe more difficult for patients with stroke in China than for those with stroke in South Korea.

A previous study found that more than 40% of a community-dwelling stroke population (compared with 9% of a control population) had limited participation in social activities and thus were at risk of social isolation, which can result in further negative health events (24). In the current study, the wheelchair group had lower CIQ, CIQ-HI, and CIQ-SI scores than the walk and walk + wheelchair groups. In addition to social and limb motor dysfunction, there are two other possible reasons for the low levels of social and family integration exhibited by the wheelchair group. First, the short duration of stroke of many of the participants in the initial assessment (the duration of stroke was no longer than 6 months in 38 participants and no more than 3 months in 24 participants) mean that they had been hospitalized since stroke onset and thus had not returned to family and society to resume their regular daily lives. Second, many participants had a reasonable level of limb motor function but these participants were worried whether they could perform daily tasks and received too much assistance from family caregivers, such that they became overly dependent on these caregivers. Alternatively, these participants were ashamed of exposing what they perceived as their “ugly” postures to relatives, friends, or neighbours and thus were reluctant to perform leisure activities such as walking. Patients with stroke who lack self-care ADL for a long period of time may fall into depression, which can adversely affect their recovery and quality of life (25–28). Therefore, patients with stroke should be encouraged to perform any daily activities they can to maintain and improve their physical motor function and psychological confidence as part of the process of rehabilitation. In addition, family members should be educated to support and encourage patients in this regard.

The productivity of the participants was generally low: only 6 out of 59 participants scored more than 0 points on the CIQ-PA. This contrasts with the findings of Doig et al. (29) and may be due to the fact that 31 of the participants in the current study were retired and 3 of the participants had been housewives before their strokes. In addition, the retired group had a lower CIQ-PA score than the employed and unemployed groups, which may be attributable to age differences. That is, a previous study found that younger people tend to be more productive than older people after acquired brain injury, with increasing age associated with lower overall and subscale scores (30). Additionally, younger people may have good support from family members or be able to re-enter post-secondary education to increase their productivity, in contrast, older people may need to be more self-motivated to achieve similar levels of productivity (31). Thus, as the participants in the current study had a mean age of 56.9 (SD = 17.0) years, and approximately 50% were retired, this may have contributed to their low level of CI.

### Predictors of Community Integration Questionnaire score

In descending order of adjusted R^2^ values, MBI score, BBS score, age, numbers of years of education, MMSE score, FMA-LE score, and FMA score were good predictors of the CIQ score at 1-year follow-up.

MBI score appears to be a crucial factor in predicting the CI level of patients with stroke. Similarly, Matos et al. reported that the MBI score was significantly associated with the CIQ score, with each 1-point increase in the MBI score resulting in an average increase of 0.168 in the CIQ score (32). People who are dependent on others for activities of daily living experience more restrictions over time than people who are independent (12). Thus, the fact that the participants in the current study were mostly dependent on others for activities of daily living underscores the importance of addressing motor impairment and improving functional independence in stroke rehabilitation to enhance patients’ CI.

BBS scores were significantly correlated with CIQ scores and were a strong predictor of CIQ scores at 1-year follow-up. This finding is consistent with the findings of Cattaneo et al (33), i.e. that there is a significant association between the CIQ score and BBS score in patients with neurological disorders. Poor balance control during walking can lead to decreased community ambulation performance, ultimately limiting stroke survivors’ participation in leisure and productive activity (31). Maintaining balance is a complex process that involves multiple factors and is commonly a problem for patients with stroke. This can increase their likelihood of falling and thus significantly decrease their quality of life, ability to perform daily tasks, and ability to reintegrate into their communities (34). The participants in the current study experienced balance problems, as the mean BBS score was 31 points at baseline, which is lower than the cut-off score, for detecting balance disorders, i.e. 40 points.

There was a significant correlation between age and the CIQ score, with age being a good predictor of the CIQ score at 1-year follow-up. Similarly, it was previously found that for every 5-year increase in age, there is an average decrease of 0.095 in the overall CIQ score (32). Moreover, older people experience more participation restrictions than younger people, possibly due to older people having higher levels of anxiety than younger people (32).

Number of years of education was significantly correlated with the CIQ score, a good predictor of the CIQ score at 1-year follow-up, and the best predictor of the CIQ-PA score at 1-year follow-up. It was previously found that education level affects CI, with a higher level of education associated with higher scores on all three CIQ subscales (35). In the current study, number of years of education was significantly related to CIQ, CIQ-HI, and CIQ-PA scores at 1-year follow-up and marginally related to the CIQ-SI score at 1-year follow-up. These findings are consistent with those of previous studies (35). However, at the initial assessment, number of years of education was significantly related to only CIQ-HI and CIQ-PA scores and was marginally related to the CIQ score. This difference may be due to the fact that the initial assessment was conducted during hospitalization, which may have weakened the impact of education on social integration. Additionally, it was previously found that more highly educated individuals exhibited a greater increase in vocational activities than less highly educated individuals, but this difference decreased over time (12).

The findings of the current study confirmed that MMSE scores are significantly correlated with CIQ scores and are a reliable predictor of CIQ scores 1 year after an initial assessment. Prior research has similarly indicated that people with impaired cognitive function tend to engage in fewer leisure and social activities than those with better cognitive functioning (12).

The findings also indicate that the impact of motor function on CI may decrease over time. FMA, FMA-UE, and FMA-LE scores were significantly correlated with CIQ, CIQ-HI, CIQ-SI, and CIQ-PA scores at the initial assessment and with CIQ-HI scores at the 1-year follow-up, but only showed marginally correlation with CIQ scores at the 1-year follow-up assessment. In addition, there was no significant correlation between motor function and the CIQ-SI or CIQ-PA at the 1-year follow-up assessment. This suggests that the participants’ initial level of motor function did not necessarily determine their future level of CI. There are two possible reasons for this. First, the motor function of patients with stroke may improve substantially after they receive stroke rehabilitation. Second, factors unrelated to physical function, such as personal factors, social factors, and relationships with professionals, can affect CI, according to a systematic review and meta-analysis (36). Therefore, it is possible that patients with stroke find ways to overcome challenges caused by stroke injuries, and that in later stages of rehabilitation the influence of factors unrelated to physical function on CI may gradually surpass the influence of physical factors on CI. This may motivate patients to actively engage in social activities, even if their motor function is not ideal.

In addition, the initial CIQ-HI score was the most predictive of CIQ, CIQ-HI, CIQ-SI, and CIQ-PA scores at 1-year follow-up. The CIQ-HI measures a person’s level of independence in activities of daily living, such as shopping, cooking, doing chores, nurturing, and gathering. These are the prerequisites for further social integration and productive activity. Thus, it is reasonable that the initial CIQ-HI score was the strongest predictor.

In summary, the participants generally had a low level of CI. Several factors, such as age, number of years of education, and the MMSE score – particularly the BBS and MBI scores – affected CIQ scores and predicted CI level at 1-year follow-up. The FMA score, including FMA-UE and FMA-LE scores, mainly affected CIQ-HI score and predicted the CI level and CIQ-HI score at 1-year follow-up. Therefore, as age and education are unmodifiable factors, a physiotherapist treating patients with stroke should focus on improving their self-care ADL, balance function, cognitive function, and upper and lower limb function, with a particular focus on self-care ADL and balance function.

### Limitations

This study has several limitations. First, the sample size was small due to the difficulty of recruiting participants during the coronavirus disease 2019 pandemic. Therefore, we could not conduct analyses according to the performance of FMA, MMSE, or MBI. Further studies should examine larger samples and patients with more uniform levels of impairment due to stroke. Second, the stroke duration of the participants varied greatly, which may have influenced the results. Thus, although there was no significant correlation between the participants’ stroke duration and CIQ scores, further research on patients with more uniform stroke duration is needed. Third, there might have been collinearity between the variables, thus care should be taken when examining the relationship between CIQ scores and the stroke-specific outcome measures. Fourth, social support and social networks were not examined; however, as these influence the progress of community reintegration (37, 38), they should be investigated in future studies. Finally, emotional factors and socioeconomic status were not investigated, and future research should explore how they affect the CI of patients with stroke.

### Conclusions

The participants in this study had a low level of CI following stroke. Age, number of years of education, and MMSE, BBS, and MBI scores were identified as factors that significantly affected CIQ scores and predicted the level of CI at 1-year follow-up. This suggests that focusing on improving balance and independence in activities of daily living during stroke rehabilitation may improve the CI of patients recovering from a stroke.
